# Predictive Role of Resilience and Hope on Adherence to Treatment in Hemodialysis Patients

**DOI:** 10.17533/udea.iee.v42n2e06

**Published:** 2024-07-04

**Authors:** Mahboobeh Magharei, Zinat Mohebbi, Sara Rostamian

**Affiliations:** 1 Master. Email: Maghareim@sums.ac.ir Shiraz University Iran Maghareim@sums.ac.ir; 2 Associate Professor, Community Based Psychiatric Care Research Center. Email: mohebbi04@yahoo.com. Corresponding author. Shiraz University Community Based Psychiatric Care Research Center Iran mohebbi04@yahoo.com; 3 Master student, Student Research Committee. Email: sararostamian70@gmail.com Shiraz University Student Research Committee Iran sararostamian70@gmail.com; 4 Department of Nursing, School of Nursing and Midwifery, Shiraz University of Medical Sciences, Shiraz, Iran. Shiraz University Department of Nursing School of Nursing and Midwifery Shiraz University of Medical Sciences Shiraz Iran

**Keywords:** renal insufficiency, chronic, renal dialysis, resilience, psychological, treatment adherence and compliance, hope., insuficiencia renal crónica, diálisis renal, resiliencia psicológica, cumplimiento y adherencia al tratamiento, esperanza., insuficiência renal crônica, diálise renal, resiliência psicológica, cooperação e adesão ao tratamento, esperança.

## Abstract

**Objective.:**

To determine the predictive role of resilience and hope on adherence to treatment in hemodialysis patients hospitalized in two hospitals affiliated to Shiraz University of Medical Sciences (Shiraz, Iran).

**Methods.:**

This is a descriptive-analytical study that was conducted in 2021-2022 on 120 patients treated in hemodialysis sections in Namazi and Shahid Faqihi teaching hospitals. Sampling was conducted using a stratified random method. Demographic information questionnaires, Connor and Davidson's resilience, Snyder's hope and adherence to kidney patients' treatment questionnaires were used to collect the data.

**Results.:**

The finds showed that the levels of resilience, hope, and adherence to treatment had hight level. More specifically, it was indicated that the mean and standard deviation for the total resilience score, the hope variable, and adherence to total treatment was 75.45±14.34, 40.43±3.66, and 80.12±18.20, respectively; which have maximum possible scores of 100, 48 and 100. Thus, it can be said that no correlation was observed between resilience and adherence to treatment variables (*p*>0.05); hope variable and adherence to treatment (*p*>0.05), and adherence to treatment with hope and resilience variables (*p*>0.05). However, hope and resilience variables showed a direct and weak correlation with each other (r=0.36, *p*<0.05); that is, patients who had more hope indicated better resilience as well.

**Conclusion.:**

Although in this study we found that the resilience and hope variables were not able to predict the treatment adherence, hope and resilience indicated a direct and weak correlation. It is recommended that nurses should pay more attention to hope and resilience of hemodialysis patients in order to promote their health.

## Resumen

**Objetivo.:**

Determinar el papel predictivo de la resiliencia y la esperanza en la adherencia al tratamiento en pacientes en hemodiálisis internados en dos hospitales afiliados a la Universidad de Ciencias Médicas de Shiraz (Shiraz, Irán).

**Métodos.:**

Se trata de un estudio descriptivo-analítico que se realizó en 2021-2022 con la participación de 120 pacientes tratados en secciones de hemodiálisis en los hospitales docentes Namazi y Shahid Faqihi. El muestreo se realizó mediante un método aleatorio estratificado. Para la recogida de datos se utilizaron datos demográficos y las escalas de resiliencia de Connor y Davidson, de esperanza de Snyder y de adherencia al tratamiento de pacientes renales.

**Resultados.:**

Los hallazgos mostraron que los niveles de resiliencia, esperanza y adherencia al tratamiento se encontraban en niveles altos. Más concretamente, se indicó que la media y la desviación estándar para la puntuación total de resiliencia, la variable esperanza y la adherencia al tratamiento total fueron 75.45±14.34, 40,43±3.66 y 80.12±18.20, respectivamente; las cuales tienen como puntajes máximos posibles 100, 48 y 100. No se observó correlación entre las variables: resiliencia y adherencia al tratamiento *(p*>0.05), esperanza y la adherencia al tratamiento (*p*>0.05), y adherencia al tratamiento y las variables esperanza y resiliencia (*p*>0.05). Sin embargo, las variables esperanza y resiliencia mostraron una correlación directa y débil entre sí (r=0.36, *p*<0.05); es decir, los pacientes que tenían más esperanza indicaron también mejor resiliencia.

**Conclusión.:**

. Aunque en este estudio encontramos que las variables resiliencia y esperanza no fueron capaces de predecir la adherencia al tratamiento, la esperanza y la resiliencia indicaron una correlación directa y débil. Se recomienda que el personal de enfermería preste más atención a la esperanza y la resiliencia de los pacientes en hemodiálisis para promover su salud.

## Resumo

**Objetivo.:**

Determinar o papel preditivo da resiliência e da esperança na adesão ao tratamento em pacientes em hemodiálise internados em dois hospitais afiliados à Universidade de Ciências Médicas de Shiraz (Shiraz, Irã).

**Métodos.:**

Trata-se de um estudo descritivo-analítico realizado em 2021-2022 com a participação de 120 pacientes atendidos nas seções de hemodiálise dos hospitais universitários Namazi e Shahid Faqihi. A amostragem foi realizada por método aleatório estratificado. Para a coleta de dados foram utilizados dados demográficos e as escalas de resiliência de Connor e Davidson, escalas de esperança de Snyder e adesão ao tratamento de pacientes renais.

**Resultados.:**

As descobertas mostraram que os níveis de resiliência, esperança e adesão ao tratamento estavam em níveis elevados. Mais especificamente, foi indicado que a média e o desvio padrão do escore de resiliência total, da variável esperança e da adesão total ao tratamento foram 75.45±14.34, 40.43±3.66 e 80.12±18.20, respectivamente; que possuem as pontuações máximas possíveis de 100, 48 e 100. Não foi observada correlação entre as variáveis: resiliência e adesão ao tratamento (*p*>0.05), esperança e adesão ao tratamento (*p*>0.05), e adesão ao tratamento e esperança e variáveis ​​de resiliência (*p*>0.05). Contudo, as variáveis ​​esperança e resiliência apresentaram correlação direta e fraca entre si (r=0.36, *p*<0.05); Ou seja, os pacientes mais esperançosos também indicaram melhor resiliência.

**Conclusão.:**

Embora neste estudo tenhamos constatado que as variáveis ​​resiliência e esperança não foram capazes de predizer a adesão ao tratamento, a esperança e a resiliência indicaram uma correlação direta e fraca. Recomenda-se que a equipe de enfermagem preste mais atenção à esperança e à resiliência dos pacientes em hemodiálise para promover sua saúde.

## Introduction

Chronic kidney disease is a complex and common disease that is rapidly growing and leads to early death and reduced quality of life, imposing a heavy burden on the health systems.^1^ Chronic kidney failure is a process of obvious and irreversible reduction in the number and function of nephrons by various mechanisms, in which toxins, fluids, and electrolytes accumulate in the body and usually ends in the final stage of kidney disease. In this case, the ability of the kidney for disposing metabolic wastes and maintaining fluids and electrolytes is lost, resulting in uremia syndrome.^2^ The prevalence of chronic kidney failure is reported to be 14.2% in the world.^3^ The population of patients with end stage renal disease is reported to be about 58,000 in Iran.^4^ The treatment plan of these patients in Iran includes kidney transplants (49%), hemodialysis (48%), and peritoneal dialysis (3%).^5^ Hemodialysis is considered as the main treatment for chronic kidney failure and although it can increase the life span of such patients, it is associated with many physical and psychological problems.^6^ Most of the patients undergoing hemodialysis suffer from mental disorders such as disturbance in social relations, anxiety, and depression.^7^ In other words, patients with kidney failure usually experience a decrease in the quality of their life, which is often caused by the long-term process of hemodialysis treatment and the complications caused by this disease.^8^ Resilience variable, as a dynamic process, helps a person's response and adaptation to stressful situations and conditions.^9^ Resilience is part of a human's ability, which enables a person to overcome the adverse conditions and use their adaptability in a proper way in order to suppress the destructive power of such situations.^10^

Hope is recognized as an influential part in the activities of a person, which is also considered as a factor that has a positive effect on sadness and lack of confidence and is a powerful tool in the fight against illness and disability.^11^ Raising hope is an effective way to improve the quality of life in people with chronic diseases and enhancing hope increases the level of self-care, quality of life and improves the general health of patients.^12^ Studies show that hemodialysis patients have a low level of hope, and hopelessness as well as the lack of meaningfulness in life can be the cause of many complications and problems for dialysis patients.^13^ Snyder *et al*. proposed the theory of hope for the first time which consisted of willpower, way-finding power, having a goal, and recognizing obstacles.^14^ Performing hemodialysis causes a change in the lifestyle, health, and roles of the hemodialysis patient. One of the problems reported in these patients is their lack of adherence to the treatment.^15^ The World Health Organization suggests the term *adherence* to be used in chronic diseases. The definition of adherence or compliance is based on the definition of this organization: the extent to which a person performs a behavior including taking medicine, following a diet, or implementing a change in lifestyle in accordance with the recommendations provided by health care personnel^.(^^16^

The level of adherence to treatment clearly affects the clinical outcomes of these patients, so that non-adherence to treatment is directly related to worse clinical outcomes. Today, there is evidence that a large number of hemodialysis patients do not adhere to the recommended treatment regimen.^17^ It can be said that in chronic diseases, including chronic kidney failure, the unfavorable consequences of non-adherence to treatment can be considered as the fundamental problems and difficulties in the health and even life of these patients. Therefore, trying to solve these issues is one of the care-treatment priorities of these patients. Considering the issue that suffering from a chronic disease and the problems caused by it can result in a lot of stress, which in turn leads to a decrease in the optimal performance and creates limitations in different parts of the patients' life. Furthermore, because hope and resilience can increase a person's power in dealing with stress, identification of effective factors for non-adherence to treatment is of special priority and importance in this regard. It can be concluded from the studies conducted in this domain,^18^^,^^19^ that high amounts of hope and resilience can help improve the quality of life and psychological factors of the patients. In addition, some related studies showed that there was a positive correlation between the quality of life and adherence to treatment.^20^ However, according to extensive investigations in various databases (Google Scholar, PubMed, etc.), no study was found so on the correlation between resilience and hope variables with adherence to treatment in hemodialysis patients. Moreover, the uncertainty of the correlation between these variables and the existence of contradictions in their correlation in various sources can be considered as one of the reasons for conducting this study. Thus, the researchers decided to carry out a study with the aim of determining the predictive role of hope and resilience on adherence to the treatment of the patient’s undergoing hemodialysis in the hospitals affiliated to Shiraz University of Medical Sciences. 

## Methods

This is an analytical-descriptive study that was conducted in 2021-2022 on the patients hospitalized in hemodialysis sections in Namazi and Shahid Faqihi teaching hospitals in Shiraz city in Fars province. After obtaining the approval of the ethics committee of Shiraz University of Medical Sciences, a list containing the names of the research population was prepared according to the list of the patients with active cases in the two selected hemodialysis sections of Shiraz University of Medical Sciences (the number of patients in the research population was 100 in Namazi hospital and 80 in Shahid Faqihi hospital). Then, sampling was performed using stratified random method. In this way, from each hemodialysis section, one hundred percent of patients eligible to enter the study and proportional to the total number of patients of that section were randomly selected as a sample. At the end, the sample of the current study consisted of 120 male and female patients with chronic kidney failure (67 patients from Namazi Hospital and 53 patients from Faqihi Hospital). To measure the sample size, we used the following formula and the Rahimi *et al.* study.^21^




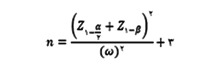




The inclusion criteria in this study were willingness to participate in the study; age between 18-60 years; hemodialysis history of at least 6 months; number of times of hemodialysis at least 2 times and at most 4 times a week; lack of suffering from chronic diseases such as advanced cardiovascular diseases like heart failure; lack of suffering from cancer, rheumatic diseases and acute pulmonary edema; absence of sensory-neural disorders such as hearing and vision impairment or cognitive disorders such as dementia; lack of suffering from mental and psychological diseases; the use of drugs in these diseases; absence of drug addiction; lack of an emotional crisis such as death of loved ones and divorce during the last 6 months. The exclusion criteria were failure to complete the questionnaire and unwillingness for further participation in the study. After obtaining the informed consent of the participants, the researchers went to the hospitals to distribute the questionnaires that should be completed by patients undergoing hemodialysis in November 2021. The stage of filling out the questionnaires lasted for three months.

After completing the questionnaires and collecting the necessary information, the data were entered into SPSS software. Data analysis included two parts: descriptive and inferential analysis. In the descriptive statistics section, mean and standard deviation were presented in the form of number and percentage. In the inferential section, research hypotheses were investigated using linear regression and Pearson's correlation coefficient. In all tests, a significance level of less than 0.05 was considered. SPSS version 24 was used for data analysis.

### Data collection instruments and process

Four questionnaires were used to collect the data in this study.

*Demographic information questionnaire*

This questionnaire was designed by the researcher in relation to the demographic and individual characteristics of the patients participating in the current study and includes information such as age, gender, education level, marital status, employment status, duration and years of dialysis, number of times of dialysis per week, and the duration of dialysis in each session.

*Connor and Davidson's resilience questionnaire*

Connor and Davidson's resilience scale was designed in 2003 by reviewing 1979-1991 research sources in the field of resilience. It consists of 25 items and its purpose is to measure the level of resilience based on the components of competence/personal strength (24-12-11-25-10-23-17-16), trust in personal instincts (20-18-15-6-7-19-14), tolerance of negative emotions (1-4-5-2-8), restraint (21-13-22), and spirituality in people (3-9). Its response range is scored on a five-point Likert scale from completely false (zero) to always true (four), and the score of this questionnaire is between 0 and 100. A score of 75-100 that is obtained from the questionnaire means a very good resilience. Connor and Davidson reported the Cronbach's alpha coefficient of resilience scale as 0.89. Furthermore, the reliability coefficient obtained from the retest method in a 4-week interval was 0.87. In the research conducted by Samani *et al.,* the reliability of this research instrument among the students was reported as 0.93 using Cronbach's alpha, and the validity was verified (using factor analysis and convergent and divergent validity) by the test makers in different normal and at-risk groups.^22^^,^^23^

*Snyder's hope scale*

This questionnaire was designed by Snyder et al. to measure hope, which consists of 12 items and two subscales of agency thinking and pathways. Scoring is based on a five-point Likert scale from completely disagree with a score of zero to completely agree with a score of four, but this scoring method is reversed for questions 3, 7, and 11 (from 4 to 0). The range of test scores is between 0 and 48, a score of 37-48 that is obtained from the questionnaire means a very good hope. Snyder *et al*. reported its overall reliability as 0.85 by retesting after 3 weeks.^14^ Grewal and Porter in 2007 described content validity as desirable.^24^ In terms of construct validity, two factors of agency thinking and pathways have been obtained through factor analysis by implementing it on 676 subjects (295 mental patients, 112 criminals and 296 students).^25^ In Kiafar et al. study (2014), the reliability obtained through Cronbach's alpha was reported as 0.80 for the entire scale, 0.68 for the agency thinking subscale, and 0.61 for the pathways, respectively.^26^

### Questionnaire of adherence to the treatment of dialysis patients

This questionnaire consists of 46 questions in 5 main sections. The first section includes general information (5 items); the second section includes the behavior of regular participation in the hemodialysis sessions (14 items). The third section includes prescribed medication consumption (9 items), the fourth section includes limitation of the fluid intake (10 items), and the fifth section includes limitation of the dietary intake (8 items). The scoring of questions No. 14, 17, 18, 26, 31, and 46 is performed using a 5-point Likert scale, which directly evaluates the treatment adherence behavior of the patients. The sum of the point of the dimensions of the treatment adherence behavior is 1200 points (participation in the hemodialysis session: 0-600 points, timely and continues use of the medicines: 200-022 points, observance of the limitation of the fluid intake: 0-200 points and observance of the limitation of the dietary intake), and the questions were graded on a Likert scale. 

The overall score of the treatment adherence is the sum of the scores of these 5 sections. The initial scores are converted into a score between 0-100. Accordingly, a score of 75-100% means a very good treatment adherence, a score of 50-74% means a good treatment adherence, a score of 26-49% means an average treatment adherence, and a score of 0-25% means a poor treatment adherence. This questionnaire was designed by Kim in 2009, who reported a favorable validity and reliability values of 83% for this instrument.^27^ The validity of this questionnaire was confirmed using face validity and in two qualitative and quantitative ways by Seyed Fatemi *et al.* Furthermore, these researchers determined the reliability of this instrument by using two methods of internal consistency and consistency reliability; also, the validity and reliability of this questionnaire was evaluated in the study of Rafiee Vardanjani *et al*. Moreover, for the items of the questionnaire, the content validity was calculated as 98%, which has a favorable score in terms of content validity. The reliability of the questionnaire was calculated as 0.85 with retesting, which was an acceptable reliability.^28^^-^^30^

## Results

The mean age of the participants in this study was 49.85 ± 12.74 years, the average duration of dialysis was 43.23 ± 8.51 months, and the average number of the sessions was 0.53 ± 2.73 sessions per week. The majority of the sample were men (55.8%), married (77.4%) with diploma education (27.5%) and retired (30%). The study samples were often completely capable of doing their daily tasks and were able to walk without any equipment (75%). (Table 1)

**Table 1:** Frequency distribution of demographic (qualitative) and clinical characteristics

Percentage	Number	Category	Variable
55.8	67	Male	Gender
44.2	53	Female	
20.8	25	Single	Marital status
77.4	93	Married	
0.8	1	Widow	
0.8	1	Divorce	
20.8	25	Illiterate	Education
26.7	32	Elementary	
17.5	21	Junior	
27.5	33	Diploma	
7.5	9	Academic	
25	30	Unemployed	Job status
34.3	41	Housekeeper	
30	36	Retires	
10.7	13	Employed	
54.1	65	Completely	The ability to do daily tasks
15	18	Relatively	
12.5	15	Somewhat	
9.2	11	A little	
9.2	11	At all	
75	90	Without the need of helping devices	Walking
10.8	13	With a cane	
4.2	5	With a walker	
10	12	With a wheelchair	
100		120	Total	

As to resilience, the mean and total standard deviation was 75.45±14.34. The highest mean in the subscales was related to trust in personal instincts with a value of 22.26±4.31, and the lowest mean in the subscales was related to tolerance of negative emotions with a value of 14.1±3.52. Moreover, 55.2% of the participants had a very good level of resilience. In terms of the dimensions of resilience, the majority of the sample (63.3%) were at a very good level in the restraint and spirituality, 51.7% were at a good level in the tolerance of negative emotions, 60.7% were at a very good level in the component of trust in personal instincts, and the majority of the 63.3% were at a good level in the component of personal competence and strength. The average and standard deviation of the total score of hope was 40.43±3.66. Moreover, in the subscale of agency thinking, the lowest average was 13.28±1.95, and the pathways indicated the highest average with a value of 13.35±2.15. In this study, 87.5% of the patients under study were at a good level in terms of hope, 60.8% were at a very good level in terms of pathways, but 44.1% were at a good level in terms of agency thinking.

As to adherence to the treatment, the mean and standard deviation of the subscale of regular participation in the hemodialysis sessions was 496.87±147.51, consumption of prescribed medication was 180.42±41.15, limitation of fluid intake was 140.41±56.25, limitation of the dietary intake was 143.75±58.54, general treatment adherence was 961.46±218.38, and total treatment adherence (transformed) was 80.12±18.20. The highest average value belonged to the subscale of regular participation in the hemodialysis sessions, and the lowest average value belonged to the subscale of the limitation of fluid intake. (Table 2(

**Table 2:** Mean, minimal and maximum values and standard deviation of treatment adherence in the hemodialysis patients

Standard deviation	Average	Maximal value	Minimal value	Variable
147.51	496.87	600	0	Regular participation in hemodialysis sessions
41.15	180.42	200	0	Prescribed drug consumption
56.25	140.42	200	0	Limitation of liquid intake
58.54	143.75	200	0	Limitation of dietary intake
218.38	961.46	1200	250	Adherence to total treatment
18.20	80.12	100	20.83	Adherence to total treatment (transformed)

68.3% of the participants obtained a very good level of total treatment adherence. Furthermore, as to the dimensions of adherence to the treatment, 40% in the limitation of the dietary intake, 31.7% in the limitation of liquid intake, 75.8% in the component of the use of the prescribed drugs, and 66.7% in the component of regular participation in hemodialysis sessions were at a very good level. Therefore, it can be mentioned that most of the participants in the current study indicated a very good treatment adherence in all dimensions. In general, 1.7% of the participants in poor level, 4.2% in average level, 25.8% in good level, and 68.3% in very good level indicated adherence to the total treatment. 

In this study, Spearman's correlation test showed that there was no correlation between resilience and adherence to the treatment (*p*>0.05, r=-0.02). It was also found that there was a strong and direct correlation between resilience and its subscales (*p*<0.05). Furthermore, there was a moderate and direct correlation between personal competence/strength with the components of trust in personal instincts, tolerance of negative emotions, restraint, and spirituality (*p*<0.05, r=0.55, r=0.33, r=0.48). The components of trust in personal instincts, restraint, and spirituality indicated a moderate and direct correlation (*p*<0.05, r=0.45). There was also a weak and direct correlation between tolerance of negative emotions and restraint and spirituality (r=00.19, *p*<0.05). Moreover, there was a weak and inverse correlation between restraint, spirituality, and hope (*p*<0.05, r=- 0.2), and there was no correlation between hope and adherence to the treatment (*p*>0.05, r=0.1). It was also found that there was a direct and moderate correlation between hope and its subscales (*p*<0.05). 

Furthermore, pathways indicated a direct and weak correlation with resilience and adherence to the treatment (*p*<0.05, r=0.32, r=0.29). It was also found that there was a strong and direct correlation between adherence to the treatment and the subscales of regular participation in the hemodialysis sessions, but there was a moderate and direct correlation with the component of prescribed medication consumption (r=0.81, r=0.53, *p*<0.05). Moreover, there was a weak and direct correlation between regular participation in hemodialysis sessions with the consumption of prescribed medicine and the restriction of dietary intake components (*p*<0.05, r=0.41, r=0.27). It was also revealed that there was a weak and direct correlation between the restriction of fluid intake and hope (r=0.19, *p*<0.05); also, a moderate and direct correlation was observed between the restriction of liquid intake and the restriction of dietary intake (*p*>0.05, r=0.6). Finally, hope and resilience showed a direct and weak correlation with each other (r=0.36, *p*<0.05). (Table 3)

**Table 3:** Correlation coefficient between treatment adherence and its subscales with resilience and hope variables

H	R	DI	FI	PM	HS	A	Variables
r=0.1 *p*=0.273	r=- 0.02 *p*=0.277	r=0.67 *p*=0.310	r=0.54 *p*= 0.111	r=0.53 *p*<0.001	r=0.81 *p*<0.001	1.00	Adherence to the treatment (A)
r=0.04 *p*=0.666	r=0.02 *p*=0.799	r=0.27 *p*=0.003	r= 0.11 *p* = 0.223	r=0.41 *p*<0.001	1.00		Participation in the hemodialysis sessions (HS)
r=0.1 *p*=0.289	r=0.1 *p*=0.314	r=0.27 *p*=0.003	r=0.3 *p*=0.001	1.00			Consumption of prescribed medicine (PM)
r=0.19 *p*=0.036	r=0.04 *p*=0.674	r= 0.6 *p*<0.001	1.00				Limitation of fluid intake (FI)
r=0.17 *p*=0.069	r=0.04 *p*=0.633	1.00					limitation of dietary intake (DI)
r=0.36 *p*<0.001	1.00						Resilience (R)
1.00							Hope (H)

A linear regression test was used to predict the effects of resilience and hope on adherence to the treatment. The results show that these two variables explain only 20% of the changes in treatment adherence (R^2^=0.20) Furthermore, it was indicated that none of these two variables could predict the treatment adherence values (*p*>0.05). In addition, the results of Spearman's correlation test showed that there was no correlation between these two variables and adherence to the treatment.

As to demographic features, Spearman's correlation test in this study showed that there was a weak and direct correlation between resilience and age (*p*<0.05). It was also indicated that there was a weak and inverse correlation between the component of trust in personal instincts and the duration of dialysis (*p*<0.05), and there was a weak and inverse correlation between hope and age (*p*<0.05). Finally, a weak and direct correlation was observed between the consumption of prescribed medicine and age (*p*<0.05).

## Discussion

This study was conducted with the aim of determining the predictive role of resilience and hope on adherence to the treatment in hemodialysis patients. In the present study, more than half of the participants were at a very good level of resilience. As in some studies, resilience level is less than present study.^18^^,^^21^ This discrepancy can be attributed to the differences in the age and type of the disease in the two studies. More patients under the study were at a good level in terms of hope. In 2016, Mirbaqher *et al.*^19^ reported lower hope in hemodialysis patients than the mean score obtained in the present study and does not agree with the results of this study. This difference can be attributed to difference in the sample size of the two studies. 

The results of the study showed a very good level of treatment adherence of the patients. In 2018, Naderi Far *et al.*^31^ reported, lower treatment adherence than the average treatment adherence score obtained in the present study. Furthermore, in 2018, Rafiee and Shafie reported in their study that the average adherence score to the total treatment was, lower than the score obtained in the present study.^15^ In 2017, Naalweh *et al.* stated that the mean score of adherence to the treatment in their study showed lower than the score obtained in the present study.^32^ Considering the reasons for high mean score of the patients' adherence to the treatment in this study compared to other studies, it can be attributed to different size of the samples, cultural differences, different trainings provided by the treatment staff and different methods of evaluating the level of adherence to the hemodialysis program in these studies. Regarding the dimensions of adherence to the treatment, the results are also different. The levels of regular participation in the hemodialysis sessions, consumption of prescribed medication, and limitation of the dietary intake were found to be very good, these levels were moderate in the study of Naderi Far *et al.* conducted in 2018.^31^ As indicated in the current study, level of these dimensions is more than the one in Naderi Far *et al*. study.^31^ There is also a possibility that, due to the passage of time, the patients’ attitude and understanding towards the disease have increased, and this has been effective in improving the level of treatment adherence.

As to determining the correlation between resilience and adherence to the treatment, the results of the current study showed that there was no correlation between resilience and adherence to the treatment in hemodialysis patients. In this regard, the results of the study by Naderi Far *et al.* showed that adherence to the therapeutic regimen had a significant effect on the quality of life of the patients. According to some previous studies, nurses can play a very important role in increasing adherence to the treatment regimen in the patients and improving the adherence conditions in the patients through establishing effective communications by the patients and supporting them.^31^Therefore, resilience and adherence to the treatment can affect a patient's quality of life and may have an indirect correlation with each other in terms of the quality of life, a subject which requires more research in this field. However, improving the level of resilience and adherence to the treatment can increase the physical condition and a patient's quality of life, which can finally lead to improvement of the level of mental health in the patient.

In terms of determining the correlation between hope and adherence to the treatment, the results of the current study showed that there was no significant correlation between hope and adherence to the treatment in patients. In the same line, the results of the study by Shareinia *et al*. in 2022 showed that there was no significant correlation between hope and adherence to the treatment regimen,^33^ a finding which is consistent with the results of this study. However, the results of the present study showed that there was a direct and weak relationship between hope and resilience. Some researchers showed in their studies that there was a significant positive relationship between hope and resilience.^34^^-^^36^These findings are consistent with the results of the present study. That is, by increasing hope, the amount of resilience increases as well. It can be mentioned that hope is a factor that enriches life and enables people to have a vision beyond their current situation, disorder, and pain. Among the positive results of improving hope, we can refer to creating a meaningful life, having energy for work; maintaining happiness and protecting life; having self-confidence, peace, and adaptability to conditions; and feeling superior in life. As to the relationship between hope and resilience, as found in this study, it is suggested that in order to improve the treatment of patients, psychological treatment programs should be used along with physical (medical) treatment programs; in this way, an attempt should be made to accelerate the recovery of the patients. 

Finally, regarding the correlation the between components and subscales, it should be mentioned that the components indicated some correlations with their subscales. It was found that there was a strong and direct correlation between resilience and its subscales, and a direct and moderate correlation was revealed between hope and its subscales. Furthermore, a strong and direct correlation was indicated between adherence to the treatment and the subscales of regular participation in the hemodialysis sessions, but it indicated a moderate and direct correlation with the consumption of prescribed medicine. In terms of demographic features, Spearman's correlation test in this study showed that there was a weak and direct correlation between resilience and age. Furthermore, it was found that there was a weak and inverse correlation between the component of trust in the personal instincts and the duration of dialysis. A weak and direct correlation existed between the use of prescribed medicine and age, and we also found a weak and inverse correlation between hope and age. It can be stated in such a way that by increasing age, the amount of hope decreases, which can be due to the fact that aging makes the conditions more difficult for old patients, resulting in some characteristics that would lead to the appearance of some behavioral habits among such patients. In their study conducted in 2022, Khachian *et al.* showed that there was a significant relationship between adherence to the treatment and demographic variables,^37^ which is consistent with the findings of the present study. One of the strengths of the present study was that no study has been conducted on the predictive role of resilience and hope on adherence to treatment in hemodialysis patients in Iran.

Limitations. One of the limitations of the current study was the lack of similar studies, which reduced the possibility of a better and more accurate comparison of the results for the researcher. As a result, it is suggested that similar studies should be conducted in order to more closely examine the impact of interventions and treatments on the hemodialysis patients by taking into account the passage of time from the disease and treatment. Furthermore, due to the lack of such studies, it is suggested that the effects of demographic, social, and clinical factors on the three components discussed in the present study should be investigated in order to gain a more comprehensive understanding of this matter. In addition, more studies are recommended to be conducted on the correlation between resilience and hope and adherence to the treatment. It is also possible that the sample size could not show this correlation statistically, thus, a similar study with a larger sample size is needed.

Conclusion. This study was conducted to investigate the predictive role of resilience and hope in adherence to the treatment of the patients who were under hemodialysis in two hospitals affiliated to Shiraz University of Medical Sciences in 2021 and 2022. The results of this study indicated that the mean scores of resilience were at the desired level. Furthermore, the average scores of hope in the study also showed that hope was at a favorable level. It was found that the level of adherence to the treatment in the present study was generally at a very good level, only in the dimension of the limitation of the fluid intake; this level was lower than other dimensions, which requires training and improving the level of awareness of these patients regarding the consequences of reducing the fluid intake in their disease. Despite the favorable levels of the three variables of resilience, hope, and adherence to the treatment, there is a need for educational interventions by nurses and medical staff to maintain and improve the cases examined in this study. Because the patient's condition may undergo physical and mental changes over time and during the treatment, which may affect these variables, it is necessary to use the guidance and advice of nurses and treatment staff. Given that hope and resilience could not predict treatment adherence in the present study, more studies are needed in this regard. 

Research implications. The main purpose of conducting a study in the field of disease and health is to apply it in different fields of the society and improve the quality of health. According to the subject of this study, in this section, the implications of the findings was discussed in the three areas of education, clinical services and management. In the field of education, it is suggested that more educational programs should be held in relation to the subject of the research because these programs can increase the level of knowledge and awareness of the performers of the interventions and treatments, which is effective in improving the implementation, results, and improving the mental and physical condition of the patients. In the field of clinical services, more attention can be paid to the role of nurses and nursing officials in holding consultation meetings and support forums to familiarize the patients with the related issues, to solve the problems and access information about the effectiveness of treatment and situation improvement, or to provide evidence from the previous studies conducted on the necessary issues for clarifying the patients, points which can be effective in improving the health condition in general. In the field of management, health managers and health policymakers can teach these concepts and their importance to health care workers during management and educational programs or through holding some workshops.

Acknowledgements. This article is extracted from a research project approved with the number of 23476 and the code of ethics (IR.SUMS.NUMIMG.REC.1400.005) on 06/19/2021 for obtaining a master’s degree in the field of nursing at Shiraz University of Medical Sciences. We hereby express our appreciation and thanks to the vice president of the research and the ethics committee of Shiraz University of Medical Sciences, the clinical research center of Namazi Hospital, as well as all the personnel of the hemodialysis department of Namazi and Shahid Faqihi Hospitals for their sincere cooperation. The authors would like to thank Shiraz University of Medical Sciences, Shiraz, Iran and also Center for Development of Clinical Research of Namazi Hospital and Dr. Nasrin Shokrpour for editorial assistance.
